# Evaluation of Host Constitutive and Ex Vivo Coccidioidal Antigen-Stimulated Immune Response in Dogs with Naturally Acquired Coccidioidomycosis

**DOI:** 10.3390/jof9020213

**Published:** 2023-02-06

**Authors:** Jared A. Jaffey, Lisa F. Shubitz, Michael D. L. Johnson, Charlotte A. Bolch, Anderson da Cunha, Ashlesh K. Murthy, Brina S. Lopez, Ross Monasky, Imani Carswell, Justine Spiker, Miranda J. Neubert, Sanjay V. Menghani

**Affiliations:** 1 Department of Specialty Medicine, College of Veterinary Medicine, Midwestern University, Glendale, AZ 85308, USA; 2 Valley Fever Center for Excellence, College of Medicine-Tucson, University of Arizona, Tucson, AZ 85724, USA; 3 Department of Immunobiology, Valley Fever Center for Excellence, BIO5 Institute, Asthma and Airway Disease Research Center, University of Arizona, College of Medicine-Tucson, Tucson, AZ 85724, USA; 4 Office of Research and Sponsored Programs, College of Graduate Studies, Midwestern University, Glendale, AZ 85308, USA; 5 Department of Pathology, College of Veterinary Medicine, Midwestern University, Glendale, AZ 85308, USA; 6 Department of Immunobiology, College of Medicine-Tucson, Tucson, AZ 85724, USA; 7 Department of Immunobiology, Medical Scientist Training Program, College of Medicine-Tucson, Tucson, AZ 85724, USA

**Keywords:** *Coccidioides* spp., cytokine, toll-like receptor, TLR2, TLR4, disseminated, pulmonary, flow cytometry, inflammation

## Abstract

The early innate immune response to coccidioidomycosis has proven to be pivotal in directing the adaptive immune response and disease outcome in mice and humans but is unexplored in dogs. The objectives of this study were to evaluate the innate immune profile of dogs with coccidioidomycosis and determine if differences exist based on the extent of infection (i.e., pulmonary or disseminated). A total of 28 dogs with coccidioidomycosis (pulmonary, n = 16; disseminated, n = 12) and 10 seronegative healthy controls were enrolled. Immunologic testing was performed immediately, without ex vivo incubation (i.e., constitutive), and after coccidioidal antigen stimulation of whole blood cultures. Whole blood cultures were incubated with a phosphate-buffered solution (PBS) (negative control) or a coccidioidal antigen (rCTS1 (105–310); 10 µg/mL) for 24 h. A validated canine-specific multiplex bead-based assay was used to measure 12 cytokines in plasma and cell culture supernatant. Serum C-reactive protein (CRP) was measured with an ELISA assay. Leukocyte expression of toll-like receptors (TLRs)2 and TLR4 was measured using flow cytometry. Dogs with coccidioidomycosis had higher constitutive plasma keratinocyte chemotactic (KC)-like concentrations (*p* = 0.02) and serum CRP concentrations compared to controls (*p* < 0.001). Moreover, dogs with pulmonary coccidioidomycosis had higher serum CRP concentrations than those with dissemination (*p* = 0.001). Peripheral blood leukocytes from dogs with coccidioidomycosis produced higher concentrations of tumor necrosis factor (TNF)-α (*p* = 0.0003), interleukin (IL)-6 (*p* = 0.04), interferon (IFN)-γ (*p* = 0.03), monocyte chemoattractant protein (MCP)-1 (*p* = 0.02), IL-10 (*p* = 0.02), and lower IL-8 (*p* = 0.003) in supernatants following coccidioidal antigen stimulation when compared to those from control dogs. There was no detectable difference between dogs with pulmonary and disseminated disease. No differences in constitutive or stimulated leukocyte TLR2 and TLR4 expression were found. These results provide information about the constitutive and coccidioidal antigen-specific stimulated immune profile in dogs with naturally acquired coccidioidomycosis.

## 1. Introduction

*Coccidioides immitis* and *C. posadasii* are dimorphic, soil-dwelling fungi that are commonly found in the southwestern United States and northern Mexico [1]. The transmission of *Coccidioides* spp. occurs primarily through inhalation of arthroconidia that are aerosolized from soil [2]. Once in the terminal bronchioles and alveoli, arthroconidia undergo spherulation and are either eliminated by the host immune response (asymptomatic infection), cause respiratory tract disease (pulmonary), or spread to extra-thoracic sites (disseminated) [2]. Similar to humans, most dogs with coccidioidomycosis have the pulmonary form of the disease, although dogs have a higher estimated incidence of dissemination (25%) compared to humans (<1%) [1,3,4].

The innate immune system includes pattern recognition receptors (PRRs), such as c-type lectin receptors (e.g., Dectin-1) and toll-like receptors (TLRs). In particular, TLR2 and TLR4 mediate fungal processing, fungal killing, and the production of cytokines and chemokines that direct adaptive responses [5,6]. The innate immune response has proven to be pivotal in antigen presentation and infection outcomes in humans and mouse models of coccidioidomycosis but has not been assessed in dogs [2,6,7]. In *Coccidioides* spp. infection, TLRs have an important role in orchestrating the recognition of spherules/endospores and initiating innate immune responses against the fungus. Toll-like receptor-2 gene expression has been shown to be upregulated in vitro in mouse peritoneal macrophages and bone marrow-derived dendritic cells stimulated with coccidioidal antigens [8]. In another study, peritoneal macrophages from TLR2^−^/^−^ mice produced less tumor necrosis factor (TNF)-α and macrophage inflammatory protein (MIP)-1 than wild-type mice [9]. Toll-like receptor-4 did not appear to affect mouse pulmonary fungal burden, survival, or humoral responses, but disseminated fungal burdens were higher in mice deficient in TLR4 [10]. Therefore, TLR4 may be important for controlling the dissemination of coccidioidomycosis [10]. The subsequent proinflammatory cytokine response after *Coccidioides* spp. recognition is necessary for the development of a Th1/Th17 cell-mediated immune response and infection control [11,12,13], while primarily anti-inflammatory (interleukin [IL]-10, IL-4) cytokine responses are associated with increased disease severity and dissemination [14,15,16,17,18].

C-reactive protein is a positive acute phase protein that is produced as a reaction to various types of systemic inflammation such as infectious, neoplastic, non-infectious inflammatory, and immune-mediated disorders [19,20,21,22,23,24,25,26,27,28,29,30,31,32]. It has been used as a viable biomarker to guide clinical decisions regarding several disorders in dogs, such as prognosis, remission, relapse, and extent of infection [26,27,30,31,32,33,34]. Evidence suggests that CRP is not only a marker of inflammation but has an active immunologic role in the host response to infections [35]. One study involving *Coccidioides* spp. showed that pretreatment of peripheral blood lymphocytes with anti-CRP antibodies reduced the killing capacity of natural killer (NK) cells for *C. immitis* in humans [36]. Collectively, this information indicates that CRP may have potential as a biomarker and a direct contributor to the host immune response to *Coccidioides* spp. infections in dogs. A preliminary investigation of CRP in dogs with coccidioidomycosis is justified before larger and more extensive studies evaluating the clinical utility and interactions with the host immune response are pursued.

Investigation into the immunopathogenesis of this disease could provide insight into the wide spectrum of clinical manifestations in dogs with coccidioidomycosis [4,37,38,39,40,41,42,43,44]. Guided by what has been learned from human clinical and mouse experimental studies, the primary objective of the study reported here was to compare constitutive and *Coccidioides* spp. antigen-stimulated leukocyte cytokine production to assess the inflammatory profile, TLR2 and TLR4 expression, and serum CRP concentrations between dogs with newly diagnosed coccidioidomycosis and seronegative healthy controls. A secondary aim was to determine whether differences in these parameters exist between dogs with pulmonary and disseminated coccidioidomycosis. We hypothesized that cytokine concentrations, serum CRP concentration, and leukocyte TLR2 and TLR4 expression would be different between dogs with coccidioidomycosis and healthy controls. We also hypothesized that there would be one or more immune parameters that differed between dogs with pulmonary and disseminated coccidioidomycosis.

## 2. Materials and Methods

### 2.1. Criteria for Selection of Cases

Client-owned dogs that had a new diagnosis of coccidioidomycosis between June 2020 and December 2020 were eligible for inclusion in this prospective cohort study. Dogs were included in the study after obtaining informed owner consent. This study was conducted in accordance with guidelines for clinical studies and approved by the Midwestern University Animal Care and Use Committee (protocol # 3000).

A diagnosis of coccidioidomycosis was made using clinical, serological, radiographic, and laboratory testing as described below. Serum antibodies against *Coccidioides* spp. were detected at commercial laboratories using either agar gel immunodiffusion (AGID) or enzyme immunoassay (EIA) (Antech Diagnostics, Irvine, CA, USA; IDEXX Laboratories, Westbrook, ME, USA; Protatek Reference Laboratory, Mesa, AZ, USA; MiraVista Labs, Indianapolis, IN, USA). Dogs were considered to have coccidioidomycosis if they had non-specific clinical signs (e.g., weight loss, decreased appetite, lethargy, fever, vomiting) with or without site-specific clinical signs, in addition to one or more of the following: positive AGID IgM or IgG, EIA IgG, direct isolation of *Coccidioides* spp. using culture, or identification of *Coccidioides* spp. organisms on cytological or histological examination. Dogs with coccidioidomycosis were categorized as pulmonary or disseminated. Requirements for the pulmonary group included signs related to respiratory tract disease (e.g., cough, increased respiratory effort, wheeze, tachypnea, cyanosis) with thoracic imaging abnormalities characteristic of *Coccidoides* spp. infection and no clinical evidence of dissemination [4,45]. Dogs in the disseminated group had confirmation or clinical suspicion of disease in one or more extrapulmonary sites. Dogs were excluded if antifungal therapy had been initiated for >7 days before enrollment.

Healthy control dogs were owned by faculty, staff, and students at the Midwestern University College of Veterinary Medicine. They were screened for enrollment if known to be in good health, had no known history of cocccidioidomycosis, and had no illnesses or medications administered, except monthly parasiticides, within 60 days of enrollment. Dogs were considered healthy based on physical examination and on a review of hematology, serum chemistry, and urinalysis results by a single board-certified small animal internist (JAJ). Control dogs were required to be seronegative for any anticoccidioidal antibodies at the time of enrollment.

### 2.2. Sample Collection

Medical records were reviewed for each dog enrolled. The age, sex, weight, and breed were recorded. The following clinical information was extracted when available: clinical signs, medications, cytological and histopathological examination reports, microbiology results, and radiographs.

Blood samples were collected into serum separator tubes and lithium heparin anticoagulant tubes. Blood was processed within 1 h of sample collection.

### 2.3. Coccidioidal Antigen Chitinase-1 Protein Preparation

The immunodominant antigen in the complex complement fixation (CF) antigen used extensively for specific detection of anticoccidioidal antibodies in humans, dogs, and other species is a 427-amino acid protein that encodes a chitinase (CTS1). Using recombinant techniques, the seroreactivity was determined to be limited to a truncated peptide between aa105-310 (rCTS1) [46], which was used in this study. rCTS1(105–310), hereinafter referred to as rCTS1, was purified as previously described [46]. Briefly, the aa105–310 truncation of rCTS1 was cloned into a Ligation Independent Cloning vector, pMCSG7, that contained a T7 promoter and N-terminal 6× His-tag, as described in [47]. The plasmid was transformed into BL21 Gold cells. The cells were grown at 37 °C in Terrific Broth to OD_600_ 0.6 and induced with 0.5 µM Isopropyl ß-D-1-thiogalactopyranoside. The temperature was reduced to 18 °C, and the cells were grown overnight (~18 h). Cells were collected and resuspended in 20 mM Tris pH 8, 200 mM NaCl, 25 mM Imidazole, and 5% glycerol with protease inhibitors and DNAse. Cells were sonicated, and the soluble fraction was run on an immobilized nickel-affinity chromatography (IMAC) (HisTrap FF, GE Healthcare). Proteins were further purified with size-exclusion chromatography (SEC) (Superdex 200, GEHealthcare) using a buffer of 20 mM Tris pH 8, 200 mM NaCl, and 5% glycerol. The protein was concentrated, and concentration was determined with absorbance at 280 nm using molecular weight and extinction coefficient. Samples at >10 μM were aliquoted into thin-walled PCR tubes and flash-frozen using liquid N_2_.

### 2.4. Serum C-Reactive Protein

Serum CRP was measured with a commercially available canine-specific sandwich ELISA (Abcam, Cambridge, UK) as previously described [48]. ELISA samples were measured in duplicate with concurrent standard curves using kit-provided canine standards; the lower limit of detection for CRP was 1.1 ng/mL. Mean absorbance was used to calculate concentration. Samples were first diluted 3× in Sample Diluent NS and then diluted 8× in Sample Diluent 25BS according to manufacturer recommendations. The optical density of the samples was determined with a Biotek Cytation 3 microplate reader (Biotek, Winooski, VT, USA) set to a wavelength of 450 nm, and background absorbance was measured at 700 nm and subtracted from sample absorbance. Sample CRP concentration was determined by plotting the kit standards using a linear curve, calculating dilute sample concentrations from this curve, and multiplying concentrations by 24 to adjust for the serial dilution.

### 2.5. Constitutive Plasma Cytokines

Whole blood collected in lithium heparin-containing tubes was centrifuged, and plasma was collected into freezer-resistant conical microcentrifuge tubes and stored at −80 °C for batch analysis. For analysis, samples were thawed, and then TNF-α, IL-6, IL-10, IL-2, IL-7, IL-8, IL-15, IL-18, granulocyte macrophage colony-stimulating factor (GM-CSF), monocyte chemoattractant protein (MCP)-1, interferon (IFN)-γ, and keratinocyte chemotactic (KC)-like were measured with a validated canine cytokine-specific multiplex bead-based assay kit (Milliplex MAP, EMD Millipore Corp, Billerica, MA, USA) as described elsewhere [49]. The median fluorescence intensity and cytokine concentration in each sample were measured in duplicate with appropriate controls and associated data analysis software (Milliplex Analyst version 5.1, EMD Millipore Corp, Billerica, MA, USA). The lower limit of detection for TNF-α, IL-6, IL-10, GM-CSF, IL-2, MCP-1, and KC-like was 48.8 pg/mL. The lower limit of detection for IL-7, IL-8, and IL-18 was 195.3 pg/mL and 9.8 pg/mL for IFN-γ.

### 2.6. rCTS1-Stimulated Leukocyte Cytokine Production

Anticoagulated whole blood was diluted 1:2 with RPMI culture medium (Thermo Fisher Scientific, Carlsbad, CA, USA) containing 200 U of penicillin/mL and 200 mg of streptomycin/mL. The blood–RPMI mixture was then transferred to 96-well plates and incubated with rCTS1 (final concentration, 10 µg/mL) or phosphate-buffered saline (PBS) as a negative control. Plates were incubated for 24 h at 37 °C in 5% CO_2_ in the dark. Following incubation, the plates were centrifuged (400× *g* for 7 min) at 21 °C. The supernatant was collected and stored at −80 °C for batch analysis of cytokines as described above.

### 2.7. Constitutive and rCTS1-Stimulated TLR2 and TLR4 Expression

Anticoagulated whole blood was diluted 1:2 with RPMI culture medium. Phycoerythrin (PE)-conjugated anti-human CD284 (TLR4, eBioscience, Inc., San Diego, CA, USA.), allophycocyanin (APC)-conjugated anti-human CD282 (TLR2, eBioscience, Inc., San Diego, CA, USA), and fluorescein isothiocyanate (FITC)-conjugated anti-canine CD45 (YKIX716.13, eBioscience, Inc, San Diego, CA, USA) were incubated with 250 µL of the blood–RPMI mixture. Antibodies were used at a dilution of 1 to 50 antibody to blood–RPMI mixture. Commercial antibodies against human TLR cross-react with canine TLR [50]. Matched isotype controls from the same manufacturer were used to eliminate background fluorescence and for optimization of the staining protocol. Samples were incubated for 30 min in the dark on ice. Cells were then washed three times with PBS and centrifuged at 600× *g* for 5 min. Next, red blood cells were lysed by adding 1 mL of ammonium–chloride–potassium (ACK) lysing buffer (155 mM NH_4_Cl, 10 mM KHCO_3_, and 0.1 mM Na_2_ ethylene diamine tetraacetic acid [EDTA]; ThermoFisher-Gibco, Waltham, MA, USA) and additionally washed twice after 30 min of incubation. Finally, cells were re-suspended in 400 µL of PBS and analyzed immediately using flow cytometry.

The blood–RPMI mixture was also transferred to two conical tubes and rCTS1 (final concentration, 10 µg/mL) was added to one tube but not the other (negative control). Both tubes were then incubated for 24 h at 37 °C in 5% CO_2_ in the dark. Following incubation, cells were washed and stained for flow-cytometric detection of TLR2 and TLR4 expression.

### 2.8. Flow Cytometry

**Figure 1:**
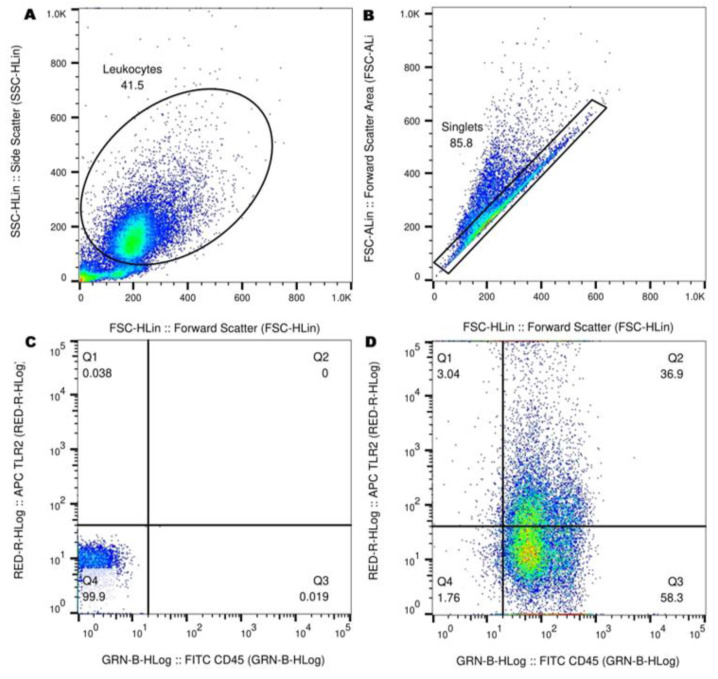
Gating of canine leukocytes using a representative sample from a healthy control. (**A**) The forward scatter-height (FSC-H) vs. side scatter-height (SSC-H) dot plot was used to define the population of interest, (**B**) followed by the exclusion of cellular debris and non-single cells (doublets, clumps) using the FSC-H vs. FSC-area (FSC-A) dot plot. (**C**) Unstained controls were used to delineate positive and negative populations, (**D**) with representative positive staining shown for CD45 and TLR2.

Constitutive and rCTS1-stimulated samples were analyzed at the Midwestern University College of Veterinary Medicine Immunology Laboratory using a Guava easyCyte 12HT (Luminex Corporation, Austin, TX, USA) and associated data analysis software (GuavaSoft 3.2, Luminex Corporation, Austin, TX, USA). A minimum of 20,000 events per sample were recorded. Forward scatter-height (FSC-H) vs. side scatter-height (SSC-H) cell size and granularity were used to define the primary population of interest: the leukocyte population. To further eliminate dead cells, debris, and cellular aggregates, singlets cells were defined using FSC-H vs. FSC-area (FSC_A). Unstained negative control samples were used to delineate positive and negative populations. The percent positive cells for both CD45 and each TLR were recorded. The representative gating methodology is shown in Figure 1.

### 2.9. Statistical Analysis

Statistical analyses were performed using proprietary software (R version 4.1.3 and SigmaPlot, Systat Software Inc., San Jose, CA, USA). Normality was assessed using the Shapiro–Wilk test or visual assessment of histogram to look at the distribution of the outcome by each group. Normally distributed data were presented as mean and standard deviation (SD), while data that were not normally distributed were presented as the median and interquartile range (IQR). Categorical data were presented as proportions. Two-sample t-tests were used for two-group comparisons of normally distributed continuous variables and Mann–Whitney rank sum tests for non-normally distributed continuous variables. Fisher’s exact test was used for categorical associations. The paired *t*-test was used for within-group comparisons of normally distributed continuous variables. When the measured cytokine or CRP concentration for the respective assay was below the lower limit of detection, data were recorded at the lower limit of detection for statistical purposes. A *p*-value of <0.05 was considered significant.

## 3. Results

### 3.1. Animal Population

Twenty-eight dogs with coccidioidomycosis were enrolled. No screened dogs were excluded. Eleven dogs were initially eligible to be included as healthy controls. One control dog was excluded because of anti-*Coccidioides* spp. IgG positivity, leaving ten healthy controls that were included in this study. There was no difference in age (*p* = 0.39), weight (*p* = 0.20), sex distribution (*p* = 0.06), or neutered status (*p* = 0.17) between dogs with coccidioidomycosis and controls. Likewise, there was no difference in age (*p* = 0.28), sex distribution (*p* = 1.00), or neutered status (*p* = 0.35) between dogs with disseminated coccidioidomycosis and those with pulmonary disease; however, dogs with disseminated disease had higher body weights (*p* = 0.03). A complete summary of demographic data can be found in Table 1.

Coccidioidomycosis was most commonly diagnosed with serology for anti-*Coccidioides* spp. antibodies alone (n = 25), or in combination with cytology (n = 2), or histopathology (n = 1). The mean rectal temperature at the visit corresponding with the diagnosis was 102.4 °F (SD, 1.2). Forty-three percent (12/28) of dogs had a rectal temperature ≥ 103 °F. The most common clinical signs included lethargy (22/28, 79%), hyporexia/anorexia (21, 75%), cough (18, 64%), lameness (8, 29%), wheeze (7, 25%), and vomit (5, 18%). Other, less commonly reported clinical abnormalities included increased respiratory effort (3, 11%), diarrhea (2, 7%), cervical pain (2, 7%), and one each of weight loss, draining tract wound, regurgitation, seizure, ataxia, and epistaxis. The estimated duration of clinical signs was available for 86% (24/28) of dogs. The median duration of clinical signs was 21 days (IQR, range; 49, 7–540 days).

Fifty-four (15/28) percent of dogs were administered one or more medications at the time of enrollment. Dogs that received medications did so for a median of 7 days (IQR, range; 2.8, 1–10 days; n = 14) before enrollment. One dog had been administered olcacitinib for approximately 360 days. Of the dogs that received medication, 53% (8/15) were administered one or more antibiotics. Non-steroidal anti-inflammatories (n = 4) and glucocorticoids (n = 2) were infrequently prescribed. Non-steroidal anti-inflammatories (carprofen, n = 3; firocoxib, n = 1) were administered for a median of 7.5 days (range, 3–8 days). One dog received prednisone per os at 0.34 mg/kg/day for 4 days preceding enrollment. Another dog was administered a single dose of dexamethasone intravenously at 0.1 mg/kg the day before enrollment. Only one dog had been administered an antifungal. This dog had received fluconazole for 3 days before enrollment.

Sixteen dogs were diagnosed with pulmonary coccidioidomycosis. Two of the sixteen dogs (13%) had positive AGID IgM results, and fourteen dogs (88%) had positive AGID IgG results. The two dogs with positive AGID IgM results also had positive AGID IgG titers. Two dogs had negative AGID IgM and IgG results but had EIA IgG positivity. The distribution of AGID IgG titers was as follows, 1:128 (n = 1), 1:64 (n = 4), 1:32 (n = 3), 1:16 (n = 3), 1:8 (n = 2), and 1:2 (n = 1). The EIA IgG results for the two dogs were 62.5 EU and 45.2 EU (>10 EU = positive result).

Disseminated coccidioidomycosis was diagnosed in 12 dogs. Eleven dogs had a single extra-thoracic organ of dissemination that included bone (n = 9) and one each of skin and pericardium. One dog had dissemination to bone and was suspected to have central nervous system involvement, but advanced imaging of the brain was not performed. Thirty-three percent (4/12) of dogs had AGID IgM positivity. All 12 dogs had positive AGID IgG titer results. The distribution of AGID IgG titers were as follows, ≥1:256 (n = 1), 1:128 (n = 1), 1:64 (n = 2), 1:32 (n = 2), 1:16 (n = 3), 1:8 (n = 2), and 1:2 (n = 1).

### 3.2. Constitutive Cytokine in Plasma of Dogs with Newly Diagnosed Coccidioidomycosis

Data from one dog with pulmonary coccidioidomycosis were excluded because of technical difficulties. Dogs with coccidioidomycosis had higher constitutive plasma KC-like concentrations (mean, SD; 162 pg/mL, 121.2) than controls (91.4 pg/mL, 53.7, t(33.6) = −2.45, *p* = 0.02). There were no differences in the remaining cytokines evaluated here (*p* > 0.05; Supplemental Table S1). No differences were found in constitutive plasma concentrations for any of the cytokines between dogs with pulmonary coccidioidomycosis and disseminated disease (*p* > 0.05 for all comparisons; Supplemental Table S2).

### 3.3. rCTS1-Stimulated Leukocyte Cytokine Production

Control dogs produced higher supernatant concentrations of TNF-α (*p* = 0.0004), IL-6 (*p* = 0.02), GM-CSF (*p* < 0.0001), IL-8 (*p* = 0.03), and IL-10 (*p* = 0.004) when whole blood was incubated with coccidioidal antigen compared with unstimulated control (i.e., PBS) (Table 2). Likewise, dogs with coccidioidomycosis produced higher concentrations of TNF-α (*p* < 0.0001), IL-6 (*p* < 0.0001), GM-CSF (*p* = 0.03), IL-8 (*p* = 0.02), IFN-γ (*p* = 0.03), KC-like (*p* = 0.01), MCP-1 (*p* = 0.01), and IL-10 (*p* = 0.0004) with coccidioidal stimulation compared with unstimulated control (Table 3).

Dogs with coccidioidomycosis produced higher coccidioidal stimulated supernatant concentrations of TNF-α (*p* = 0.003), IL-6 (*p* = 0.04), IFN-γ (*p* = 0.03), MCP-1 (*p* = 0.02), IL-10 (*p* = 0.02), and lower IL-8 (*p* = 0.003) than controls (Table 4). Subgroup comparisons of dogs with pulmonary and disseminated coccidioidomycosis revealed no differences in coccidoidal-stimulated cytokine concentrations (Supplemental Table S3).

### 3.4. Constitutive and rCTS1-Stimlated Leukocyte TLR2 and TLR4 Expression

Data from six dogs with coccidioidomycosis (pulmonary, n = 4; disseminated, n = 2) were excluded from the analysis of TLR2 and TLR4 expression because of technical difficulties. This resulted in data from 32 total dogs being available for evaluation (pulmonary, n = 12; disseminated, n = 10; controls, n = 10). There were no differences in constitutive or rCTS1-stimulated leukocyte expression of TLR2 or TLR4 between dogs with coccidioidomycosis and controls (*p* > 0.05; Table 5). Moreover, neither constitutive nor rCTS1-stimulated leukocyte expression of TLR2 and TLR4 differentiated pulmonary from disseminated dogs (*p* > 0.05; Supplemental Table S4).

### 3.5. C-Reactive Protein

**Figure 2:**
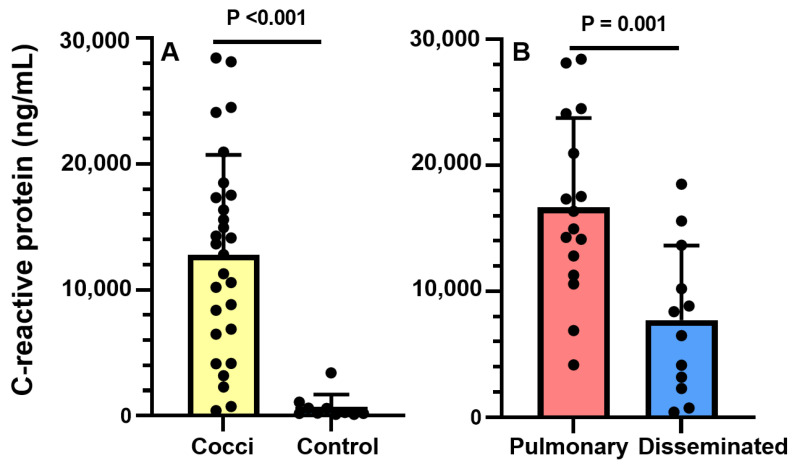
Comparison of serum C-reactive protein (CRP) concentrations between dogs with (**A**) coccidioidomycosis (n = 28) and healthy controls (n = 10) and (**B**) those with pulmonary (n = 16) and disseminated (n = 12) disease. Bars and error bars represent the mean and standard deviation, respectively. Enclosed circles represent individual data points. Dogs with coccidioidomycosis (mean, SD; 12,822 ng/mL, 7917) had higher serum CRP concentrations than controls (679.09 ng/mL, 1008.75, t(29) = −7.94, *p* < 0.001). Dogs with pulmonary coccidioidomycosis (mean, SD; 16,659.51 ng/mL, 7091.89) had higher serum CRP concentrations than those with disseminated disease (7705.33 ng/mL, 5939.99, t(26) 3.63, *p* = 0.001).

Dogs with coccidioidomycosis had higher serum CRP concentrations than controls (*p* < 0.001; Figure 2A). Similarly, dogs with pulmonary coccidioidomycosis had higher serum CRP concentrations than those with disseminated disease (*p* = 0.001; Figure 2B).

## 4. Discussion

Cytokine signatures produced by innate immune cells characterize important defensive responses mediated by both the innate and adaptive immune systems [6]. A 12-plex cytokine panel is as rich an assessment as can be made in dogs with commercially available kits. Using this assay, dogs in this study with active coccidioidomycosis had higher constitutive plasma KC-like concentrations compared to healthy controls. The primary function of KC-like (also known as CXCL1 and growth-related oncogene-α) is to induce neutrophil chemotaxis and activation [51]. This chemokine has not been investigated in coccidioidomycosis but is typically higher in other infectious diseases compared to non-infectious inflammatory disorders and healthy controls across various species including dogs [52,53,54,55,56,57]. The migration of neutrophils from circulation into tissues is required for the containment of infections including coccidioidomycosis; however, subsequent excessive release of oxidants and proteases from neutrophils can lead to substantial tissue injury [58,59,60]. Therefore, it is unknown if higher plasma KC-like concentrations would convey clinical advantages or disadvantages in dogs with coccidioidomycosis. One example that demonstrates that higher KC-like concentrations could be advantageous in a fungal infection comes from a murine model of invasive aspergillosis in which transient overexpression of KC in the lungs improved survival and decreased fungal burden in infected mice [61]. Our results indicate that future research is warranted to elucidate the immunologic role and kinetics of KC-like expression in dogs with coccidioidomycosis. Moreover, this chemokine could prove to be a useful prognostic biomarker when measured at baseline evaluation, as demonstrated in dogs with pyometra and *Babesia canis* [52,62].

There were no differences in the remaining 11 constitutive plasma cytokine concentrations between dogs with coccidioidomycosis and controls. In addition, there were no differences in any of the evaluated plasma cytokines in dogs with pulmonary versus disseminated disease. There is limited published information regarding constitutive blood cytokine concentrations in humans with coccidioidomycosis at the time of diagnosis. One study found that patients with coccidioidomycosis had higher serum IL-6, IL-18, IL-12, and IL-10 than controls [63], while another reported no difference in constitutive TNF-α, IL-1β, or IL-6 concentrations in supernatant from patients with active coccidioidomycosis and healthy controls [64]. The latter study did not indicate if samples were obtained at the time of diagnosis or if antifungal therapy had been instituted before sample acquisition. One possible explanation for the lack of differences in expected proinflammatory cytokines in our study and others is that coccidioidomycosis is a dynamic infection with projected temporal changes in the inflammatory milieu. Though blood was collected at the time of diagnosis in our study, it is unknown when the dogs were infected. Another point to consider is that it is possible the multiplex assay used in this study was relatively insensitive to detect small changes in cytokine concentrations in dogs. While the multiplex assay is widely used, some cytokine concentrations are commonly below the limits of detection regardless of health status in both humans and dogs [52,54,65,66,67,68].

To assess more disease-specific immune responses, we evaluated the ex vivo release of cytokines in the supernatant after whole blood was incubated with a coccidioidal antigen. The process by which immune cells are challenged with coccidioidal antigen ex vivo offers a different perspective of the host immunological response [69]. Stimulation with rCTS1 induced the production of some cytokines above background in both healthy controls and dogs with coccidioidomycosis as highlighted in Table 2 and Table 3, respectively. Understanding the reasons for increased cytokine production in healthy controls was beyond the scope of this exploratory study, but one possible explanation is that despite no historical evidence of coccidioidomycosis, a dog residing in this endemic area may have been previously infected and we do not have a test to accurately determine this. A second possibility is that rCTS1 is an inflammatory peptide recognized by some cells in the whole blood. A more specific assay looking at separated mononuclear cells might yield different results, as we could query the T-cell responses specifically. In addition, comparing healthy dogs from the endemic area to dogs from outside the endemic area and specific-pathogen-free dogs might also help determine whether the responses to rCTS1 are non-specific or a marker of subclinical exposure to coccidioidal antigens. This could be a future investigation.

When comparing stimulated cytokine concentrations of ill dogs to healthy controls, dogs with coccidioidomycosis produced higher stimulated concentrations of TNF-α, IL-6, IFN-γ, IL-10, MCP-1, and lower IL-8 compared to controls, indicating there is a cytokine signature associated with the *Coccidioides* spp. infection in sick dogs. The greater magnitude of ex vivo release of proinflammatory cytokines after coccidioidal antigen stimulation is also seen in humans with active coccidioidomycosis compared to controls [11]. Collectively, these results establish a baseline understanding of cytokines that may be important in the immune response to *Coccidioides* spp. infection in dogs with active coccidioidomycosis. Confirmatory studies might also determine if this is an immunosignature for active coccidioidomycosis in dogs.

In contrast to our hypothesis, the cytokine results showed no differences between dogs with pulmonary and disseminated disease. There is limited published information regarding comparisons of ex vivo stimulated cytokine production in humans with coccidioidomycosis based on the extent of infection (disseminated versus pulmonary) and healthy controls. One study found no difference in supernatant concentrations of TNF-α, IL-6, or IL-1β between healthy patients and individuals with pulmonary or disseminated coccidioidomycosis [64]. However, that study only investigated three cytokines and had a relatively small sample size. While not statistically significant (*p* = 0.12), it is interesting to note that dogs in our study with disseminated disease produced an average of 4.6 times less IFN-γ than dogs with only pulmonary involvement following stimulation with coccidioidal antigen. Deficiencies in IFN-γ or its signaling pathway are risk factors for disseminated coccidioidomycosis in humans [14,16,17,70]. Therefore, additional investigation with a larger sample size and possibly a separated mononuclear cell population is warranted to determine whether similar derangements surrounding IFN-γ are indicative of dissemination in dogs with coccidioidomycosis. Ideally, this would be supported by allelic investigations surrounding potential defects in the IFN-γ/IL-12 pathway. Dogs experience a much higher incidence of disseminated disease compared to humans, and it would be a leap forward to understand if dogs have a functional predisposition toward dissemination [1,4].

Because of the role of TLR2 and TLR4 shown in the immune pathways and infection outcomes in mice [8,9,10], we were somewhat surprised to observe no differences in TLR2 and TLR4 in these dogs. However, the lack of differences in expression in the peripheral blood cells of dogs with and without coccidioidomycosis may have several explanations. To begin with, mouse models are well-controlled in terms of genetics, timing and dose of infection, and the ability to measure disease outcomes such as fungal burden and lung cell parameters using a collection of entire tissues. Client-owned dogs are heterogeneous in breed background and time from infection to onset of illness. In addition, they cannot be subjected to invasive or destructive procedures and, thus, we are largely limited to using peripheral blood as a sample, similar to studies in humans. A second difference between the majority of published data on mouse models is the use of either live or dead whole organisms or glucan–chitin particles for stimulation of collected cells [8]. In this study, we used a serologically immunodominant recombinant protein antigen. It is possible that rCTS1 does not signal through TLR2 or TLR4. Ampel demonstrated that PBMCs from humans with coccidioidomycosis produced lower concentrations of TNF-α when TLR2 and TLR4 were blocked with antibody during stimulation with the coccidioidal extract, T27K, a complex glycoprotein antigen mixture. Results from that study highlight that other antigens may have a role in TLR2 and TLR4 responses and the rCTS1 may not best elicit this [71]. It would be interesting to stimulate dog cells with glucan–chitin particles and more complex coccidioidal antigens, and then reassess the TLR2 and TLR4 expression. Finally, working with a more controlled population of cells from the peripheral blood of dogs might show responses of individual cell populations that are drowned in the milieu of total peripheral blood cells.

Dogs with coccidioidomycosis had higher serum CRP concentrations than controls. Similarly, serum CRP concentrations were higher in dogs with pulmonary disease compared to those with dissemination. These results are in contrast with findings from our previous small case-control study that found serum CRP concentration could not differentiate pulmonary from disseminated coccidioidomycosis in dogs [72]. These conflicting results may be attributed to differing disease characteristics between the two populations and small sample sizes in both studies. Dogs with pulmonary coccidioidomycosis in the current study had higher serum CRP concentrations and anticoccidioidal IgG titers compared to dogs with pulmonary disease in the previous study. Serum CRP concentrations are commonly elevated in humans with coccidioidomycosis; however, there have been no published studies that have investigated its clinical value as a biomarker [73,74,75,76,77]. The specific reason dogs in our study with pulmonary disease had higher serum CRP concentrations than those with dissemination is unknown and likely multifactorial. One possible explanation is that dogs with disseminated disease may have infectious foci located in immunoprivileged sites (e.g., eye or central nervous system) or within sequestered granulomas that limit antigenic exposure and systemic inflammation. Ours was a preliminary investigation of CRP in dogs with coccidioidomycosis and thus its role in the host immune response was not explored. C-reactive protein binds to phosphocholine, a common cell wall component of many microorganisms, including fungi [78]. This binding activates complement and opsonizes pathogens for phagocytosis. It also ligates Fcγ receptors on the surface of leukocytes, which increases phagocytic capacity and the release of inflammatory cytokines [78]. Taken together, CRP has intriguing potential not only as a biomarker but also as a component in the host immune response in dogs with coccidioidomycosis. Collectively, these results provide the rationale for future studies to investigate the clinical utility and biological activity of CRP in dogs with coccidioidomycosis.

Our study had several limitations that must be considered. Dissemination could have been present in some dogs that were classified as having the pulmonary form of disease. Definitive exclusion of dissemination in client-owned dogs is impractical, as it would require necropsy data or extensive and costly imaging and invasive sampling procedures. The inclusion of healthy control dogs was purposefully strict because we wanted to focus on the immune response of dogs with novel exposure. However, we cannot definitively rule out that healthy control dogs were subclinically exposed to *Coccidioides* spp. if they reside in the endemic area, as noted above. To address this would require enrolling healthy controls from non-endemic locations. The methodologies used in this study to assess immune profiles were intentionally broad in order to allow cellular reactions to occur in a more physiologic milieu, potentially enhancing the clinical relevance of the ex vivo results. However, it is possible that different results would have been gathered if cytokine production and TLR expression were evaluated in individual immune cell types. We utilized a single incubation time (i.e., 24 h) for the ex vivo coccidioidal antigen stimulation phase of this study based on similarly designed studies in mice and humans [8,64,79,80,81]. Likewise, blood samples were incubated with coccidioidal antigen at one concentration (i.e., 10 µg/mL), which was chosen based on the authors’ experience. Different results may have been gathered if different coccidioidal incubation concentrations and times were utilized. In addition, the antigen used was a single recombinant polypeptide with no sugar moieties. The results might have been more similar to mouse and human studies if a more complex antigen was used to stimulate the dog cells. In unpublished studies, the complex antigens appeared to kill the cultured peripheral blood mononuclear cells from dogs, and more work must be performed to optimize this assay. Approximately half of the dogs with coccidioidomycosis had been administered one or more drugs before enrollment. Most of these dogs were administered an antibiotic but some received short courses of anti-inflammatory drugs. The immune profile in some of these dogs might have been affected by these medications. This is a limitation of utilizing dogs with naturally acquired coccidioidomycosis that need their clinical illness addressed, sometimes even while awaiting specific diagnostic test results. Due to the exploratory nature of the comparisons in this study, statistical tests were conducted without the adjustment for multiplicity, which is a limitation. The adjustment for multiple testing ensures a stricter penalty on false-positive rates that controls the overall type 1 error rate for all the statistical comparisons. In many basic science experiments that are exploratory and not confirmatory, investigators are able to conduct the additional statistical tests of interest without controlling for multiple testing but need to recognize the possibility of false-positive results [82]. Lastly, our sample size was relatively small and might have contributed to not detecting differences that actually exist between dogs with pulmonary and disseminated coccidioidomycosis, in particular the pattern of IFNγ production between the two groups.

## 5. Conclusions

In conclusion, the current study provides information about the constitutive and ex vivo coccidioidal antigen-stimulated immune cytokine profile in dogs with naturally acquired coccidioidomycosis. Dogs with coccidioidomycosis had higher constitutive plasma KC-like concentrations compared to controls. Following stimulation with rCTS1, dogs with coccidioidomycosis developed a proinflammatory cytokine profile consistent with a specific response to *Coccidioides* spp. Differences in the immune response between dogs with pulmonary and disseminated coccidioidomycosis were not detected with these assays. Serum CRP concentrations were higher in dogs with coccidioidomycosis than controls, indicating that all dogs enrolled in this study at the time of diagnosis exhibited systemic inflammation, and in contrast to the other parameters, it was higher in dogs with pulmonary disease. Lastly, no differences in constitutive or coccidioidal antigen-stimulated leukocyte expression of TLR2 or TLR4 were identified. The interesting new insights from this investigation provide a platform for further refinement and development of assays to evaluate anticoccidioidal immune responses in naturally infected dogs.

## Figures and Tables

**Table 1:** Demographic data for 28 dogs with coccidioidomycosis and 10 healthy control dogs.

	Coccidioidomycosis (n = 28)		Controls (n = 10)
Variable	Pulmonary (n = 16)	Disseminated (n = 12)	
Age (years) ^a^	6.7 (8.1)	4.0 (2.5)	6.0 (4.8)
Weight (kg) ^b^	15.7 (8.8)	23.6 (8.8)	14.8 (7.4)
Male/female	8, 8	6, 6	1, 9
Neutered/intact	14, 2	8, 4	10, 0
Breed (n)	MBD (12), Border Collie (1), Cavalier King Charles Spaniel (1), Beagle (1), Rhodesian Ridgeback (1)	MBD (5), Golden Retriever (2), Boxer (1), German Shepherd (1), Australian Shepherd (1), Pit Bull Terrier (1), Bluetick Coonhound (1)	MBD (6), Chihuahua (1), Australian Shepherd (1), Shepherd (1), Miniature Schnauzer (1)

kg, kilogram; n, number; MBD, mixed breed dog. ^a^ Data presented as median (interquartile range). ^b^ Data presented as mean (standard deviation).

**Table 2:** Comparison of supernatant cytokine concentrations in 10 healthy controls after exposure to phosphate-buffered solution (negative control) and stimulated with rCTS1 antigen. Data presented as mean and standard deviation.

Cytokine (pg/mL)	Stimulated (rCTS1 Antigen)	Unstimulated (PBS)	*p*-Value
TNF-α	1136.2 (396.6)	428.2 (340.1)	0.0004
IL-6	226.5 (105.5)	112.3 (74.7)	0.02
IL-10	1218.8 (713.9)	251.3 (186.5)	0.004
IFN-γ	11.8 (6.3)	9.8 (0.0)	0.34
GM-CSF	204.2 (92.6)	68.1 (36.5)	<0.0001
IL-2	69.1 (57.8)	67.8 (50.2)	0.64
IL-7	195.3 (0.0)	195.3 (0.0)	--
IL-8	7345.6 (2136.1)	6004.2 (3346.6)	0.03
IL-15	195.7 (1.4)	195.3 (0.0)	0.34
KC-like	452.7 (157.6)	378.1 (146.6)	0.16
IL-18	195.3 (0.0)	195.300 (0.0)	--
MCP-1	2223.9 (1088.7)	1935.5 (632.7)	0.18

Tumor necrosis factor (TNF), interleukin (IL), interferon (IFN), granulocyte macrophage colony-stimulating factor (GM-CSF), keratinocyte chemotactic (KC), monocyte chemoattractant protein (MCP), data not available (--).

**Table 3:** Comparison of supernatant cytokine concentrations in 28 dogs with coccidioidomycosis after stimulation of peripheral blood cells with rCTS1 antigen or unstimulated in phosphate-buffered solution (negative control). Data presented as mean and standard deviation.

Cytokine (pg/mL)	Stimulated (rCTS1 Antigen)	Unstimulated (PBS)	*p*-Value
TNF-α	3411.8 (2834.9)	975.3 (1911.8)	<0.0001
IL-6	418.8 (440.8)	227.0 (443.1)	<0.0001
IL-10	2448.1 (2321.1)	817.9 (1320.2)	0.0004
IFN-γ	327.6 (721.8)	19.0 (29.4)	0.03
GM-CSF	981.1 (2025.7)	256.3 (405.9)	0.03
IL-2	123.0 (183.3)	116.9 (148.6)	0.51
IL-7	262.3 (237.5)	253.3 (181.4)	0.45
IL-8	4493.2 (2426.3)	3211.7 (2623.0)	0.02
IL-15	348.2 (492.5)	333.0 (397.6)	0.48
KC-like	427.9 (184.8)	282.2 (196.6)	0.01
IL-18	245.9 (187.4)	236.4 (144.2)	0.28
MCP-1	5056.7 (5750.0)	2555.5 (1974.1)	0.01

Tumor necrosis factor (TNF), interleukin (IL), interferon (IFN), granulocyte macrophage colony-stimulating factor (GM-CSF), keratinocyte chemotactic (KC), monocyte chemoattractant protein (MCP).

**Table 4:** Comparison of rCTS1 antigen-stimulated supernatant cytokine concentrations in 28 dogs with coccidioidomycosis and 10 healthy controls. Data presented as mean and standard deviation.

Cytokine (pg/mL)	Coccidioidomycosis	Control	*p*-Value
TNF-α	3411.8 (2834.9)	1136.2 (396.6)	0.0003
IL-6	418.8 (440.8)	226.5 (105.5)	0.04
IL-10	2448.1 (2321.1)	1218.8 (713.9)	0.02
IFN-γ	327.6 (721.8)	11.8 (6.3)	0.03
GM-CSF	981.1 (2025.7)	204.2 (92.6)	0.05
IL-2	123.0 (183.3)	69.1 (57.8)	0.18
IL-7	262.3 (237.5)	195.3 (0.0)	0.15
IL-8	4493.2 (2426.3)	7345.6 (2136.1)	0.003
IL-15	348.2 (492.5)	195.7 (1.4)	0.11
KC-like	427.9 (184.8)	452.7 (157.6)	0.69
IL-18	245.9 (187.4)	195.3 (0.0)	0.16
MCP-1	5056.7 (5750.0)	2223.9 (1088.7)	0.02

Tumor necrosis factor (TNF), interleukin (IL), interferon (IFN), granulocyte macrophage colony-stimulating factor (GM-CSF), keratinocyte chemotactic (KC), monocyte chemoattractant protein (MCP).

**Table 5:** Comparison of constitutive and rCTS1 antigen-stimulated leukocyte expression of toll-like receptor (TLR)-2 and TLR4 in 22 dogs with coccidioidomycosis and 10 healthy controls. Data presented as mean and standard deviation.

Percent Positive Leukocytes	Coccidioidomycosis	Control	*p*-Value
**Constitutive**			
CD45+/TLR2+ (%)	13.3 (10.0)	17.1 (12.2)	0.41
CD45+/TLR4+ (%)	7.7 (9.5)	2.4 (6.6)	0.08
**rCTS1 antigen stimulated**			
CD45+/TLR2+ (%)	16.5 (17.7)	16.7 (10.7)	0.97
CD45+/TLR4+ (%)	2.3 (3.0)	3.5 (4.3)	0.43

## Data Availability

The data presented in this study are openly available in the Kaggle repository. https://www.kaggle.com/datasets/jaredjaffey/canine-coccidioidomycosis-immunology, accessed on 4 February 2023.

## Supplementary Materials

The following supporting information can be downloaded at: https://www.mdpi.com/article/10.3390/jof9020213/s1, Table S1: Comparison of constitutive plasma cytokines in 27 dogs with coccidioidomycosis and 10 healthy controls. Table S2: Comparison of constitutive plasma cytokine concentrations in 15 dogs with pulmonary and 12 dogs with disseminated coccidioidomycosis. Table S3: Comparison of rCTS1 antigen-stimulated supernatant cytokine concentrations in 16 dogs with pulmonary coccidioidomycosis and 12 with disseminated disease. Table S4: Comparison of constitutive and rCTS1 antigen-stimulated leukocyte expression of toll-like receptor (TLR)-2 and TLR4 in 12 dogs with pulmonary coccidioidomycosis and 10 dogs with disseminated disease.
