# Supplementary material for: Evaluation of Host Constitutive and Ex Vivo Coccidioidal Antigen-Stimulated Immune Response in Dogs with Naturally Acquired Coccidioidomycosis
Source: J Fungi (Basel). 2023 Feb 6;9(2):213. doi: 10.3390/jof9020213 (PMC9959558; doi:10.3390/jof9020213)
Supplement: Supplementary file 1 [file jof-09-00213-s001.zip › Supplemental Table S1 VF Immune.docx]

Supplemental Table S1. Comparison of constitutive plasma cytokines in 27 dogs with coccidioidomycosis and 10 healthy controls. Data presented as mean and standard deviation.

| **Cytokine (pg/mL)** | **Coccidioidomycosis** | **Control** | **P-value** |
| --- | --- | --- | --- |
| TNF-α | 102.2 (146.6) | 90.0 (126.8) | 0.81 |
| IL-6 | 127.9 (231.6) | 94.9 (145.1) | 0.61 |
| IL-10 | 56.1 (23.8) | 52.4 (11.4) | 0.53 |
| IFN-γ | 17.5 (34.1) | 9.8 (0.0) | 0.25 |
| GM-CSF | 250.4 (626.4) | 225.6 (527.3) | 0.91 |
| IL-2 | 270.8 (618.8) | 226.3 (448.4) | 0.81 |
| IL-7 | 492.7 (1001.3) | 420.8 (711.6) | 0.81 |
| IL-8 | 747.0 (1117.0) | 575.8 (694.2) | 0.58 |
| IL-15 | 667.1 (1402.2) | 552.0 (1094.7) | 0.80 |
| KC-like | 162.0 (121.2) | 91.4 (53.7) | 0.02 |
| IL-18 | 378.6 (627.6) | 195.3 (0.0) | 0.14 |
| MCP-1 | 244.7 (182.4) | 271.2 (136.7) | 0.64 |

Tumor necrosis factor (TNF), interleukin (IL), interferon (IFN), granulocyte macrophage colony-stimulating factor (GM-CSF), keratinocyte chemotactic (KC), monocyte chemoattractant protein (MCP)
