# Supplementary material for: Evaluation of Host Constitutive and Ex Vivo Coccidioidal Antigen-Stimulated Immune Response in Dogs with Naturally Acquired Coccidioidomycosis
Source: J Fungi (Basel). 2023 Feb 6;9(2):213. doi: 10.3390/jof9020213 (PMC9959558; doi:10.3390/jof9020213)
Supplement: Supplementary file 1 [file jof-09-00213-s001.zip › Supplemental Table S2 VF Immune.docx]

Supplemental Table S2. Comparison of constitutive plasma cytokine concentrations in 15 dogs with pulmonary and 12 dogs with disseminated coccidioidomycosis. Data presented as mean and standard deviation.

| **Cytokine (pg/mL)** | **Pulmonary** | **Disseminated** | **P-value** |
| --- | --- | --- | --- |
| TNF-α | 103.4 (139.6) | 100.7 (161.1) | 0.96 |
| IL-6 | 132.2 (249.9) | 122.6 (217.5) | 0.92 |
| IL-10 | 60.0 (31.0) | 51.2 (8.3) | 0.31 |
| IFN-γ | 22.0 (45.6) | 12.0 (6.4) | 0.42 |
| GM-CSF | 293.3 (782.0) | 196.8 (378.7) | 0.68 |
| IL-2 | 299.2 (692.9) | 235.3 (539.8) | 0.79 |
| IL-7 | 550.9 (1181.9) | 420.0 (762.6) | 0.73 |
| IL-8 | 901.2 (1446.5) | 554.3 (461.5) | 0.39 |
| IL-15 | 658.2 (1370.1) | 678.3 (1502.5) | 0.97 |
| KC-like | 180.3 (139.4) | 139.2 (94.6) | 0.37 |
| IL-18 | 419.4 (761.8) | 327.6 (432.9) | 0.70 |
| MCP-1 | 279.8 (218.6) | 200.8 (118.4) | 0.24 |

Tumor necrosis factor (TNF), interleukin (IL), interferon (IFN), granulocyte macrophage colony-stimulating factor (GM-CSF), keratinocyte chemotactic (KC), monocyte chemoattractant protein (MCP)
