# Supplementary material for: Evaluation of Host Constitutive and Ex Vivo Coccidioidal Antigen-Stimulated Immune Response in Dogs with Naturally Acquired Coccidioidomycosis
Source: J Fungi (Basel). 2023 Feb 6;9(2):213. doi: 10.3390/jof9020213 (PMC9959558; doi:10.3390/jof9020213)
Supplement: Supplementary file 1 [file jof-09-00213-s001.zip › Supplemental Table S3 VF Immune.docx]

Supplemental Table S3. Comparison of rCTS1 antigen-stimulated supernatant cytokine concentrations in 16 dogs with pulmonary coccidioidomycosis and 12 with disseminated disease. Data presented as mean and standard deviation.

| **Cytokine (pg/mL)** | **Pulmonary** | **Disseminated** | **P-value** |
| --- | --- | --- | --- |
| TNF-α | 3958.3 (2534.9) | 2683.1 (3154.4) | 0.26 |
| IL-6 | 522.2 (533.5) | 281.0 (229.3) | 0.12 |
| IL-10 | 2685.5 (2628.3) | 2131.5 (1900.5) | 0.52 |
| IFN-γ | 493.1 (920.0) | 107.0 (177.4) | 0.12 |
| GM-CSF | 1097.0 (2545.0) | 826.6 (1093.3) | 0.71 |
| IL-2 | 123.0 (197.5) | 123.1 (171.1) | 1.00 |
| IL-7 | 257.7 (244.9) | 268.6 (237.7) | 0.91 |
| IL-8 | 4712.3 (2428.4) | 4201.1 (2499.1) | 0.59 |
| IL-15 | 294.0 (310.1) | 420.5 (673.9) | 0.56 |
| KC-like | 480.6 (178.3) | 357.8 (176.2) | 0.08 |
| IL-18 | 250.0 (218.7) | 240.5 (144.7) | 0.89 |
| MCP-1 | 6066.5 (6820.3) | 3710.2 (3776.3) | 0.26 |

Tumor necrosis factor (TNF), interleukin (IL), interferon (IFN), granulocyte macrophage colony-stimulating factor (GM-CSF), keratinocyte chemotactic (KC), monocyte chemoattractant protein (MCP)
