# Supplementary material for: Evaluation of Host Constitutive and Ex Vivo Coccidioidal Antigen-Stimulated Immune Response in Dogs with Naturally Acquired Coccidioidomycosis
Source: J Fungi (Basel). 2023 Feb 6;9(2):213. doi: 10.3390/jof9020213 (PMC9959558; doi:10.3390/jof9020213)
Supplement: Supplementary file 1 [file jof-09-00213-s001.zip › Supplemental Table S4 VF Immune.docx]

Supplemental Table S4. Comparison of constitutive and rCTS1 antigen-stimulated leukocyte expression of toll-like receptor (TLR)-2 and TLR4 in 12 dogs with pulmonary coccidioidomycosis and 10 dogs with disseminated disease. Data presented as mean and standard deviation.

|  | **Pulmonary** | **Disseminated** | **P-value** |
| --- | --- | --- | --- |
| **Constitutive** | | | |
| CD45+/TLR2+ (%) | 12.6 (10.0) | 14.2 (10.4) | 0.72 |
| CD45+/TLR4+ (%) | 10.5 (10.3) | 4.3 (7.5) | 0.12 |
| **rCTS1 antigen stimulated** | | | |
| CD45+/TLR2+ (%) | 13.4 (15.1) | 20.2 (20.5) | 0.40 |
| CD45+/TLR4+ (%) | 1.6 (2.0) | 3.0 (3.9) | 0.32 |
